# Design of a Temporally Augmented Text Messaging Bot to Improve Adolescents’ Physical Activity and Engagement: Proof-of-Concept Study

**DOI:** 10.2196/60171

**Published:** 2024-10-10

**Authors:** Adrian Ortega, Christopher C Cushing

**Affiliations:** 1 Center for Behavior Intervention Technologies Department of Preventive Medicine Northwestern University Feinberg School of Medicine Chicago, IL United States; 2 Clinical Child Psychology Program The University of Kansas Lawrence, KS United States; 3 Schiefelbusch Institute for Life Span Studies The University of Kansas Lawrence, KS United States

**Keywords:** digital intervention, youth, exercise, SMS, mHealth, augmented text messaging, bot, adolescents, adolescent, physical activity, engagement, reliability, physical activity intervention, digital health, digital support, community

## Abstract

**Background:**

Digital interventions hold promise for improving physical activity in adolescents. However, a lack of empirical decision points (eg, timing of intervention prompts) is an evidence gap in the optimization of digital physical activity interventions.

**Objective:**

The study examined the feasibility and acceptability, as well as the technical and functional reliability, of and participant engagement with a digital intervention that aligned its decision points to occur during times when adolescents typically exercise. This study also explored the impact of the intervention on adolescents’ moderate to vigorous physical activity (MVPA) levels. Consistent with the Obesity-Related Behavioral Interventions Trials (ORBIT) model, the primary goal of the study was to identify opportunities to refine the intervention for preparation for future trials.

**Methods:**

Ten adolescents completed a 7-day baseline monitoring period and Temporally Augmented Goal Setting (TAGS), a 20-day digital physical activity intervention that included a midday self-monitoring message that occurred when adolescents typically start to exercise (3 PM). Participants wore an accelerometer to measure their MVPA during the intervention. Participants completed questionnaires about the acceptability of the platform. Rates of recruitment and attrition (feasibility), user and technological errors (reliability), and engagement (average number of text message responses to the midday self-monitoring message) were calculated. The investigation team performed multilevel models to explore the effect of TAGS on MVPA levels from preintervention to intervention. In addition, as exploratory analyses, participants were matched to adolescents who previously completed a similar intervention, Network Underwritten Dynamic Goals Engine (NUDGE), without the midday self-monitoring message, to explore differences in MVPA between interventions.

**Results:**

The TAGS intervention was mostly feasible, acceptable, and technically and functionally reliable. Adolescents showed adequate levels of engagement. Preintervention to intervention changes in MVPA were small (approximately a 2-minute change). Exploratory analyses revealed no greater benefit of TAGS on MVPA compared with NUDGE.

**Conclusions:**

TAGS shows promise for future trials with additional refinements given its feasibility, acceptability, technical and functional reliability, participants’ rates of engagement, and the relative MVPA improvements. Opportunities to strengthen TAGS include reducing the burden of wearing devices and incorporating of other strategies at the 3 PM decision point. Further optimization of TAGS will inform the design of a Just-in-Time Adaptive Intervention for adolescent physical activity and prepare the intervention for more rigorous testing.

## Introduction

### Background

Physical activity is a vital health behavior to prevent the onset of cardiovascular disease, diabetes, cancer, and obesity [1]. Adolescents exhibit low rates (<10%) of meeting the physical activity guidelines, 60 minutes of moderate to vigorous physical activity (MVPA) per day [2]. The trend toward physical inactivity during adolescence confers risk for future disease [3-5]. Therefore, interventions to increase and sustain exercise during adolescence are needed to improve physical activity and mitigate these risks.

However, the impact of physical activity interventions for adolescents is small; effect sizes for objectively assessed physical activity interventions translate to approximately a 4-minute change in daily MVPA postintervention [6,7]. Digital intervention delivery may serve as an opportune modality for improving the impact of physical activity interventions due to the capabilities of technology to provide just-in-time (in-the-moment) and contextually tailored support to users [8,9].

However, most digital interventions have failed to engage the user, especially adolescents [10]. High “nonusage” attrition and a failure to implement digital tools in real-world settings are factors known to undermine the impact of digital interventions [10-14]. Optimal engagement with digital tools for adolescents is likely hindered by the unattuned design of most digital interventions to meet the goals, preferences, and needs of adolescents across moments and contexts [10,14]. Greater intervention precision, such as enhanced adaptation and tailoring of message timing and content, may improve digital intervention engagement.

Digital support is best leveraged during moments when users are receptive to performing health behavior change and engaging with a digital intervention. Within a Just-in-Time Adaptive Intervention (JITAI) framework, the moment when a digital intervention is delivered is known as a “decision point” [15]. Intervening at empirically supported times when a user is likely to be receptive (eg, engage with the intervention or exhibit the target behavior) can improve engagement and health outcomes and minimize the waste of a digital message [15]. A systematic review [16] revealed that most physical activity JITAIs modified decision points to occur at interventionist-defined times [17-19] or at user-defined times [20]. For example, Bond et al [17], Pellegrini et al [18], and van Dantzig et al [19] delivered prompts to take breaks from sedentary behavior at interventionist-defined lengths of uninterrupted sedentary time. Finkelstein et al [20] only sent messages to individuals to take sedentary breaks during user-preferred times. The aforementioned studies integrated useful decision points based on reasonable logic such as when users were exhibiting excessive health-compromising behaviors or at times when users choose to receive messages; however, these decision points were not informed by empirical evidence, such as when the users has or have shown to be receptive to digital support. Therefore, the lack of empirical decision points embedded within digital interventions indicates a gap in the design of physical activity JITAIs as intervening at critical moments when users have demonstrated to be receptive would theoretically improve engagement with the intervention and the target behavior. In addition, Hardeman et al [16] noted that most JITAIs lacked evidence-based behavior change techniques (BCTs) for improving physical activity. Therefore, incorporation of empirically identified decision points for delivering digital support is an opportunity to promote greater engagement.

### Network Underwritten Dynamic Goals Engine

**Figure 1:**
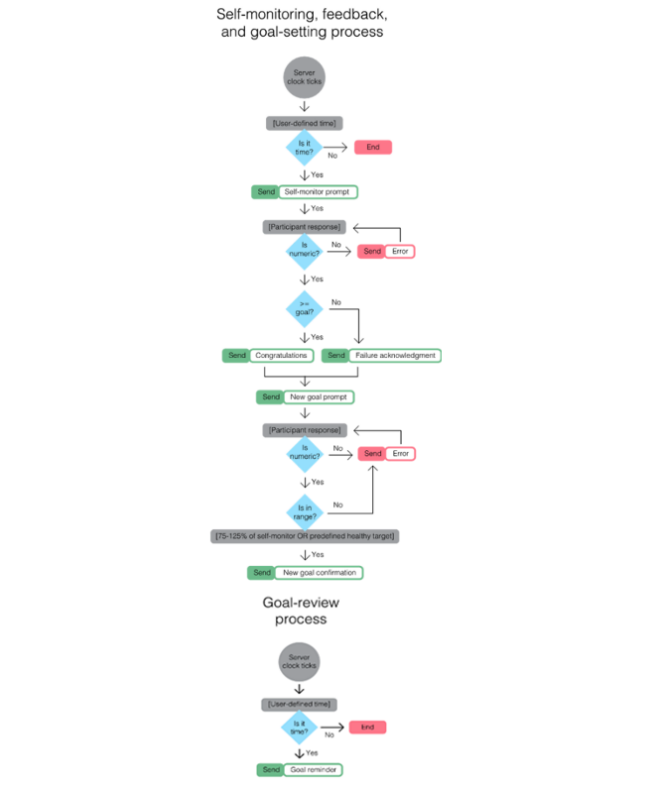
Visualization of the Network Underwritten Dynamic Goals Engine (NUDGE) platform process which depicts the flow of the NUDGE’s decision points and decision rules.

The Network Underwritten Dynamic Goals Engine (NUDGE) [21] is a text message–delivered physical activity intervention (Figure 1) for adolescents based on Cybernetic Control Theory and empirically supported BCTs for improving physical activity in youth and adults (self-monitoring, feedback, goal setting, and goal review) [22-25]. A pilot study of NUDGE demonstrated large improvements on MVPA [21]. Additional optimization of NUDGE to fit the needs of the user across moments and contexts could further improve NUDGE’s efficacy. There is evidence to suggest that adolescents are most likely to meet their typical levels of MVPA between the hours of 5 PM and 8 PM based on accelerometer data [26]. Therefore, providing digital support before or during the hours of 5 PM and 8 PM could maximize the opportunity that adolescents reach their MVPA goals before the likelihood of meeting their exercise goals diminishes after 8 PM [26]. Realistically, sending digital support to improve exercise should occur slightly earlier, around 3 PM, to allow adolescents to dedicate time toward exercising.

### Temporally Augmented Goal Setting

The Obesity-Related Behavioral Interventions Trials (ORBIT) model provides a sequence of phases for initially designing and later optimizing behavioral treatments [27]. This model emphasizes refinement of interventions in the design phase to maximize the efficiency of the intervention while preserving intervention strength. Testing distinct components of interventions in the early phases of the ORBIT sequence is necessary to distill the essential aspects of the intervention [27]. Therefore, the evaluation of optimum decision points is critical for informing the development of a future adolescent physical activity JITAI.

As part of our ongoing efforts to iteratively strengthen the NUDGE intervention [21,28] and design a JITAI to improve adolescent MVPA, we designed a Temporally Augmented Goal Setting (TAGS) physical activity intervention that aligned the timing of NUDGE’s texts to occur during moments when adolescents have empirically demonstrated that they are likely to exercise [26]. The main difference between the NUDGE and TAGS is that TAGS provides an additional opportunity for self-monitoring when adolescents enter the window of opportunity for exercising at 3 PM to help adolescents reach their physical activity goals at an opportune moment. This study tested the feasibility, acceptability, and the technical and functional reliability of the TAGS intervention. We also evaluated rates of participant engagement with the TAGS intervention and explored the impact of TAGS on MVPA. This study was a proof-of-concept study situated in phase IIa of the ORBIT model [27] with the goal of determining whether TAGS’s timing of support merits additional testing in future efficacy trials as demonstrated by adequate feasibility, acceptability, technical and functional reliability, and user engagement.

### Study Aims and Hypotheses

The first aim of the study was to test the feasibility, acceptability, and the technical and functional reliability of TAGS, as well as to examine participants engagement with the texts. The investigation team hypothesized that TAGS would be feasible as indicated by a recruitment rate above 80% and an attrition rate less than 20% of recruited participants. The investigation team also hypothesized that TAGS would be acceptable as indicated by average scores above the midpoint on the acceptability surveys. The investigation team examined the technical and functional reliability of the TAGS by calculating the prevalence of errors with the TAGS technology. The investigation team hypothesized that the engagement rate with the TAGS messages would be greater than 50%, given that this was the average engagement rate in the NUDGE trial [21].

As part of exploratory analyses to test the impact of TAGS on MVPA, the investigation team explored changes from preintervention to intervention levels of MVPA in the TAGS group. In addition, the team explored differences in MVPA between the TAGS and NUDGE interventions. These analyses were considered exploratory given the small TAGS sample, the quasi-experimental design for comparing the TAGS and NUDGE groups, and the fact that the main purpose of the study was to determine opportunities to refine the TAGS protocol for future trials.

## Methods

### Ethical Considerations

All study procedures were approved by The University of Kansas institutional review board (STUDY00145685). All participants provided informed consent and assent. The data included in this study were deidentified and hosted on a secure, encrypted server. Participants earned up to US $50 for study participation (US $10 for attending each of the 3 study visits, US $10 for wearing the accelerometer for 24 hours on 18 out of 20 intervention days, and US $10 for returning the study equipment).

### Participants

The investigation team recruited 10 adolescents from the Greater Kansas City area to complete the TAGS protocol from 2021 to 2022. Recruitment strategies included flyering in community areas, social media posts, and invitations to participants from other studies who consented to receive study advertisements. Eligibility criteria included adolescents who (1) were between the ages of 13 and 18 years, (2) lived at home with a legal caregiver, (3) were able to read at a fifth-grade level in English, (4) owned a smartphone, (5) were free from any physical conditions that would limit their physical mobility, and (6) were free from any significant vision concerns such as vision loss or low vision. The investigation team excluded adolescents from the TAGS intervention who previously participated in the NUDGE study [21].

As part of this study’s exploratory analyses, the 10 TAGS participants were matched to 10 participants who completed the NUDGE intervention [21] in 2017. The investigation team matched TAGS participants to NUDGE participants by demographic variables: sex assigned at birth, age, and race (with priority given in this order). The protocols for both studies were very similar (eg, identical inclusion and exclusion criteria, same accelerometers, and participating in the intervention for the same amount of time).

### NUDGE Condition

As outlined by Cushing et al [21], after enrollment in NUDGE, a participant selected a physical activity goal. At a user-defined time the next morning, the NUDGE bot sent a message reminding the user of their goal. Later in the same day, at another user-defined time, participants were prompted to self-monitor their physical activity. The participants replied to the text with a numerical value representing their physical activity attainment. This value was compared against their goal. The NUDGE bot then provided feedback to the user indicating whether their goal was met and requested a new goal. The NUDGE bot accepted goal values only between 75% and 125% of their last physical activity value, so that new goals were not set too high or too low to be useful. This goal was then stored and this process repeated daily for 20 days.

### TAGS Procedures

#### Informed Consent and Assent Process

Caregivers and adolescents provided their informed consent and assent, respectively. The investigation team then mailed study devices to participants and scheduled a virtual baseline visit.

#### Baseline Visit

Participants completed study measures. The investigation team then oriented the adolescent to the accelerometer and provided instructions for wear use. The investigation team instructed participants to wear the accelerometer for 7 days to collect a baseline assessment of their physical activity. The investigation team then scheduled the TAGS orientation visit to occur at least 7 days following the baseline assessment.

#### TAGS Digital Intervention Orientation Visit

At the TAGS orientation visit, which lasted approximately 10 minutes, the investigation team demonstrated how to respond to the TAGS texts. Participants then selected an initial physical activity goal between 15 and 60 minutes.

#### 20-Day TAGS Intervention

The TAGS intervention operated in the same fashion as NUDGE. At a predetermined time the next morning, the TAGS bot sent a message reminding the user of their goal. However, later in the day at 3 PM, they were prompted to self-monitor how much physical activity they have accrued *thus far* (eg, “How many minutes of exercise have you done so far?”). Their response was compared against their goal value. TAGS then provided quantitative feedback regarding how many minutes of physical activity they have left to meet their goal (eg, “You only have 10 more minutes of exercise left to meet your goal!”). At 8 PM, they were prompted to self-monitor their total minutes of physical activity attainment throughout the day. The TAGS bot then provided feedback to the user indicating whether they met their goal and requested a new goal. This process repeated daily for the 20-day intervention period. Similar to the NUDGE intervention, participants self-reported their physical activity in the texts because active engagement in the control theory process (ie, comparing one’s progress to their proposed goal) is important for goal attainment [21]. Although self-report of physical activity would be less accurate than an accelerometer, self-report allows adolescents to more thoughtfully engage in self-monitoring, which is one of the most effective BCTs for physical activity [25].

#### GPSs and Ecological Momentary Assessment Data Collection

During the intervention period, participants also wore a GPS tracker (BT-Q1000XT, Qstarz International Co) to measure their location. In addition, participants completed ecological momentary assessment (EMA) surveys 4 times per day. The GPS and EMA data were collected as secondary data for future analyses.

#### Exit Visit

Participants completed a virtual exit visit to complete the study’s acceptability and usability surveys. Participants returned study devices via mail. The investigation team provided financial compensation to participants according to the specifications communicated to them during the consent or assent process.

### Measures

#### Demographics

Participants self-reported their demographics (age, sex assigned at birth, gender, race, ethnicity, and approximate family income) on an electronic survey.

#### Feasibility

Feasibility was defined as the rate of recruitment of eligible participants and rate of attrition of recruited participants. The investigation team also recorded reasons for adolescents’ ineligibility or declined participation to identify potential barriers of the TAGS protocol.

#### Acceptability

The investigation team administered 3 measures as evidence for acceptability (Multimedia Appendix 1). The first measure was an adapted 8-item survey based on the Consumer Satisfaction Questionnaire (CSQ-8) assessing overall participant satisfaction with TAGS. The CSQ-8 is a reliable and valid instrument with adolescents [29]. The Cronbach a value for the CSQ-8 within the TAGS sample was 0.94.

Participants additionally completed the 10-item System Usability Scale (SUS), which is a measure of participants’ enjoyment, ability to use, and comprehend the TAGS intervention technology. The SUS is a very popular usability measure given its short length, high reliability, and convergent validity with other usability measures [30]. The Cronbach a value for the SUS within the TAGS sample was 0.90.

Finally, participants completed a general acceptability survey to assess the level of burden of the study. The measure was developed by the investigation team’s lab and comprises 5 items scored on a 7-point Likert scale and 2 open-ended items regarding what participants liked and did not like about the study. The investigation team coded responses to the open-ended items to generate frequencies of common themes about participants’ likes and dislikes of TAGS. The investigation team did not measure the internal consistency for this survey as the 5 quantitative items measured burden related to several different aspects of the study that might not be consistent with each other.

#### Technical and Functional Reliability

The investigation team calculated errors in the TAGS platform as well as common user errors such as the number of unexpected messages received from participants (eg, participant engaged with the TAGS intervention at the wrong time), invalid messages received from participants (eg, participant responded to the TAGS bot in the incorrect format), out-of-bounds goal setting (eg, participant set a goal outside of the predefined range), and occasions of “frustration” when the user continued to respond incorrectly to the TAGS system despite TAGS correction feedback.

#### Engagement

User engagement was defined dichotomously as replying (1) or not (0) to the TAGS message (eg, 3 PM self-monitoring message) before the next digital prompt.

#### Physical Activity

Participants wore the ActiGraph wGT3X-BT accelerometer (ActiGraph LLC) to measure MVPA. The investigation team instructed participants to wear the accelerometer for 24 hours per day on their nondominant wrist during the entire study duration. The investigation team processed accelerometer data using the Actilife software (version 6.10.2; ActiGraph LLC). Using 60-second epoch files, the investigation team applied activity algorithms to valid wear days, defined as greater than 10 hours of wear per day. Nonwear and sleep periods were identified using the Troiano and the Sadeh algorithms, respectively, and removed [31,32]. The investigation team then applied the Chandler algorithm to calculate minutes of MVPA [33]. The Chandler cut points were modified to adjust for the 60-second epoch data. An identical accelerometer processing method has been used in several studies [21,26,34].

### Data Analytic Plan

#### Aim 1

#### Feasibility

The investigation team determined rates of recruitment and attrition.

#### Acceptability

We calculated average participant responses on the surveys using descriptive statistics.

#### Technical and Functional Reliability

The team calculated prevalence of errors with the TAGS technology.

#### Engagement

The investigation team removed the first day of message data to account for different participant start times, which would affect the number of messages received across participants. The investigation team then calculated the percentage of participant engagement with the TAGS message.

#### Exploratory Aims: Physical Activity

The investigation team used multilevel modeling procedures to explore the impact of TAGS on preintervention to intervention MVPA. The investigation team estimated this model using a full-information maximum likelihood estimator in SAS PROC MIXED. The data were structured so that days were nested within participants. Each TAGS participant was expected to have approximately 7 days of baseline physical activity data and 20 days of physical activity data while on intervention. The dependent variable (minutes of daily MVPA) was first entered into a model with no predictors to allow for the computation of an intraclass correlation coefficient (ICC). A fixed effect of study phase (intervention=1, baseline=0) was then entered into the model to estimate these changes from preintervention to intervention and an effect size was calculated. The investigation team calculated the effect size of preintervention to intervention changes by dividing the adjusted mean difference of MVPA between time points (ie, fixed parameter estimate of the study phase variable) by the pooled SD.

Another model compared the differences in MVPA between the TAGS and NUDGE intervention groups. The NUDGE group did not have a baseline monitoring period; therefore, the investigation team was unable to compare the effect of TAGS to NUDGE accounting for baseline physical activity. Instead, the investigation team modeled the effect of time by fitting a variable reflecting intervention day (ranging from 0 to 19) centered at the first day of intervention as done in the NUDGE study [21]. Similar to the previous models, the ICC was first calculated. Next, the investigation team modeled the effect for time (ie, days across the intervention period). Group membership was then entered into the model as a predictor with the reference group centered at zero (eg, TAGS=1, NUDGE=0). The effect size of TAGS (relative to NUDGE) was calculated by dividing the adjusted mean difference of physical activity between groups by the pooled SD between groups. The investigation team classified effect size estimations as small (*d*=0.2), medium (*d*=0.5), and large (*d*=0.8) based on Cohen [35] criteria.

## Results

### Baseline Demographics and Descriptive Statistics

Demographics for the 10 TAGS participants as well as for the 10 matched NUDGE participants are shown in Table 1. More males than females were recruited in the TAGS sample compared with the NUDGE sample so that one of the TAGS males was matched to a NUDGE female. Seven participants were in high school, 2 were attending college, and 1 was in middle school.

**Table 1:** Demographics of adolescent participants included in TAGSa and NUDGEb.

		TAGS intervention group (n=10)	NUDGE intervention group (n=10)
**Sex Assigned at Birth, n (%)**			
	Male	6 (60)	5 (50)
	Female	4 (40)	5 (50)
**Gender,** **n (%)**			
	Cisgender man/boy	6 (60)	NAx^c^
	Cisgender woman/girl	3 (30)	NAx
	Transgender man/boy	1 (10)	NAx
	Transgender woman/girl	0 (0)	NAx
Age at baseline (years), mean (SD)		15.4 (2.07)	15.3 (2.16)
**Race** **, n (%)**			
	White or Caucasian	7 (70)	9 (90)
	Asian	3 (30)	0 (0)
	Hispanic/Latino	0 (0)	1 (10)
	Other/Multiracial	0 (0)	0 (0)
**Approximate family income (US $)** **, n (%)**			
	≤60,000	1 (10)	1 (10)
	>60,000	8 (80)	9 (90)
	Missing	1 (10)	0 (0)

^a^TAGS: Temporally Augmented Goal Setting.

^b^NUDGE: Network Underwritten Dynamic Goals Engine.

^c^NAx: not assessed.

### Aim 1

#### Feasibility

Seventeen adolescents were screened and all were eligible to complete TAGS. Three eligible adolescents never responded to complete the informed consent process. Two other eligible adolescents elected not to enroll due to concerns about wearing the study devices. Twelve adolescents enrolled in the study leading to a 70.6% (12/18) recruitment rate, failing to meet the ≥80% threshold. Two participants withdrew after consenting leading to an attrition rate of 16.7% (2/12), meeting the ≤20% threshold. These participants also had concerns about wearing the study devices and withdrew before the baseline visit. Zero participants withdrew after starting the TAGS intervention.

#### Acceptability

The mean item response on the CSQ-8 was 3.11 (SD 0.61) on a scale with a possible range of 1-4 (higher values reflect greater satisfaction), meeting the hypothesized threshold. This suggests that participants, on average, reported satisfaction with the TAGS intervention on the CSQ-8. The mean item response on the SUS was 4.20 (SD 0.67) on a scale with a possible range of 1-5 (higher values reflect greater usability). This indicates that participants, on average, reported an ability to easily use as well as comprehend the TAGS intervention technology. This finding also met the hypothesized threshold. In addition, participants reported a general acceptability above the hypothesized threshold for the overall TAGS protocol (mean 5.24, SD 1.00) on a scale with a possible range of 1-7 (higher values reflect greater acceptability), indicating good acceptability of the study on average. Common themes from open-ended survey questions regarding participants’ “likes” about the TAGS intervention were the ease and convenience of TAGS to everyday life (eg, “it was not intrusive to everyday life,” n=4), their maintenance of physical activity (eg, “it helped me stay active on a daily basis,” n=4), the TAGS prompts or reminders (eg, “...to complete some exercise that I would probably not have done without the constant reminders,” n=2), the friendliness of the TAGS intervention (eg, “the friendly feel to it,” n=2), and the ability to contribute to a scientific study (n=1). Some participants disliked wearing the accelerometer on their wrist (n=5) due to its size, appearance, and (or) discomfort. Another participant said the “texts were easy to miss.” Other dislikes of the study were related to other components of the TAGS protocol, such as the amount of EMA surveys to complete (n=4), forgetting to carry the GPS (n=2), charging the GPS (n=1), and not enough representative response options for items on the EMA surveys (n=1).

#### Technical and Functional Reliability

There were no issues with the TAGS messaging system when participants were on intervention. All TAGS messages were sent on time and successfully delivered messages to participants phones. There were 3 instances when a user attempted to engage with the intervention at the wrong time. Only 1 user responded to the TAGS bot using an invalid format (eg, responding with “30 minutes” instead of “30”); this occurred 7 times. There were 4 occasions across users when a user set an out-of-bounds goal that was below or above the predefined range provided by the TAGS bot. During each of these instances, the user was able to select an appropriate goal within the predefined range after receiving correction feedback from the TAGS bot. However, there were 3 occasions of frustration when a single user continued to incorrectly respond to the TAGS bot despite correction feedback.

#### Engagement

**Figure 2:**
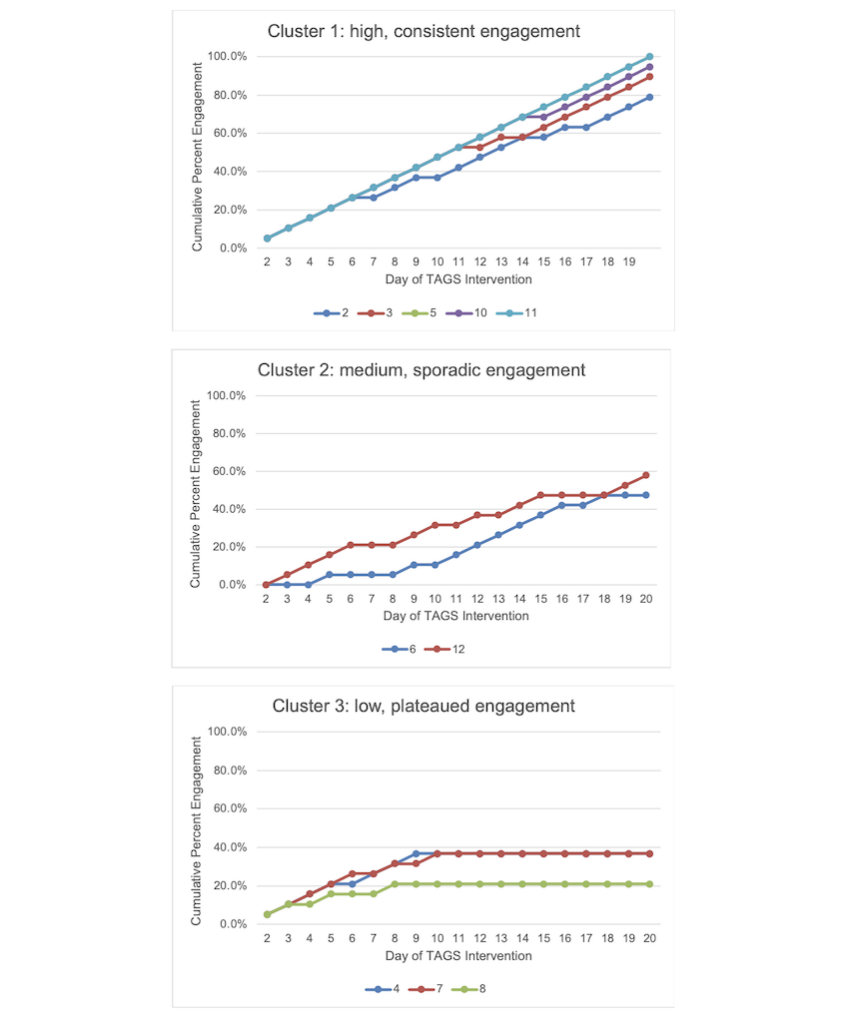
Clustered illustrations of cumulative participant engagement with TAGS message at 3 PM across the duration of the intervention. Colored lines represent a participant’s trajectory of cumulative engagement with the TAGS message at 3 PM across intervention days. Plateaus in these lines indicate patterns of nonengagement. Cluster 1 depicts participants with high levels of engagement who continued to respond to the message throughout the intervention. Cluster 2 depicts participants with medium levels of engagement with mixed patterns of responding to the message throughout the intervention. Cluster 3 depicts participants with low levels of engagement who stopped responding to the message after approximately the first week of intervention. TAGS: Temporally Augmented Goal Setting.

The average engagement with the TAGS message at 3 PM was 66.3% (12.6/19 days), successfully meeting the ≥50% hypothesis for engagement participation. However, there was substantial variability in overall participant engagement with this message as shown in Table 2. Table 2 also depicts the engagement rates for when participants responded to the self-monitoring message that occurred at the end of the day as well as the message to set a new goal for the following day. To further examine individual variability in trajectories of participant engagement with the TAGS message, the investigation team calculated participants’ percentage of cumulative engagement with the message of the intervention (computed as follows: [cumulative number of replied messages / 19] × 100). The investigation team then sorted participants into clusters based on their overall percentage and patterns of engagement throughout the intervention. Figure 2 illustrates these 3 clusters. Cluster 1 depicts 5 participants with high engagement (≥75% overall engagement) who consistently responded (<2 consecutive days of nonresponding) to the TAGS message throughout the intervention. Cluster 2 depicts 2 participants with medium levels of engagement (overall engagement between 40% and 75%) with mixed patterns of responding (≥2 consecutive days of nonresponding) to the TAGS message throughout the intervention. Cluster 3 depicts 3 participants with low engagement (<40% overall engagement) and stopped responding to the TAGS message after approximately the first week of intervention. These 3 participants all shared concerns about device burden and discomfort.

**Table 2:** Patterns of engagement with (responding to) the 20-day TAGSa bot prompts by participants.

Participant	Engagement days^b^	Percent engagement^c^, %	Self-monitored days^d^	Percent self-monitored^e^, %	Goal-set days^f^	Percent goal set^g^, %
TAGS 002	15	78.9	17	89.5	15	88.2
TAGS 003	17	89.5	18	94.7	17	94.4
TAGS 004	7	36.8	18	94.7	16	88.9
TAGS 005	19	100.0	19	100.0	18	94.7
TAGS 006	9	47.4	11	57.9	8	72.7
TAGS 007	7	36.8	7	36.8	6	85.7
TAGS 008	4	21.1	7	36.8	6	85.7
TAGS 010	18	94.7	19	100.0	18	94.7
TAGS 011	19	100.0	19	100.0	18	94.7
TAGS 012	11	57.9	9	47.4	8	88.9
Overall^h^	12.6	66.3	14.4	75.8	13	90.2

^a^TAGS: Temporally Augmented Goal Setting.

^b^Days when the participant replied to TAGS message at 3 PM before the next digital prompt.

^c^The number of engagement days divided by 19.

^d^Days when the participant replied to the overall self-monitoring message at the end of the day before the next digital prompt.

^e^The number of self-monitored days divided by 19.

^f^The days when participants set a new goal for the following day after self-monitoring.

^g^The number of goal-set days divided by the number of self-monitored days.

^h^Averages across participants.

### Exploratory Aims

#### MVPA Changes From Preintervention to Intervention Within TAGS

The empty model for MVPA indicated that approximately 59% of the variance in MVPA was between-person and 41% was within-person. Based on the final model (Table 3), the average TAGS participant obtained about 33.0 minutes of MVPA before beginning the intervention as indicated by the intercept. The main effect of study phase was not significant but indicated that the average TAGS participant obtained 2.32 minutes more of MVPA during the TAGS intervention compared with the preintervention period. The effect size of the model implied means was *d*=0.05 (small effect).

**Table 3:** Final models comparing the effect of TAGS with NUDGEa and preintervention on moderate to vigorous physical activity.

Fixed effects	TAGS versus NUDGE^b^				TAGS intervention versus TAGS preintervention^c,d^			
	*B*	Lower limit of 95% CI for slope	Upper limit of 95% CI for slope	*P* value	*B*	Lower limit of 95% CI for slope	Upper limit of 95% CI for slope	*P* value
Intercept	46.1	—^e^	—	—	33.0	—	—	—
Time (day)^f^	0.08	–0.31	0.48	.68	—	—	—	—
Group/phase	–12.4	–46.7	22.0	.46	2.32	–5.62	10.3	.57

^a^TAGS: Temporally Augmented Goal Setting. NUDGE: Network Underwritten Dynamic Goals Engine. This model was conditioned such that the intercept represents the number of moderate to vigorous physical activity (MVPA) minutes for a NUDGE participant on the first day of the study, with group coded as TAGS = 1 and NUDGE = 0.

^b^Random effects: intercept 1296.1 (SE 444.4); residual: 721.4 (SE 51.8).

^c^This model was conditioned such that the intercept represents the number of MVPA minutes for a TAGS participant during the preintervention period, with the intervention phase coded as 1 and preintervention phase coded as 0.

^d^Random effects: intercept 1058.4 (SE 5144.4); residual: 746.3 (SE 71.1).

^e^Not available.

^f^Time reflects day of intervention and ranges from 0 to 19.

#### MVPA Differences Between TAGS and NUDGE

In an empty model for MVPA the ICC was 0.64, indicating that approximately 64% of the variance in MVPA was between-person and 36% was within-person. In the models for time, a fixed linear model of time fits best and was retained for subsequent analyses. Based on the final model (Table 3), the average NUDGE participant obtained about 46.1 minutes of MVPA during the intervention as indicated by the intercept. The main effect of group was not significant but indicated that the average TAGS participant obtained 12.4 minutes fewer of MVPA than the average NUDGE participant. This mean difference relative to the pooled SD between groups translates to a Cohen *d* effect size of –0.28.

## Discussion

This proof-of-concept study examined the feasibility, acceptability, and technical and functional reliability of and adolescent engagement with a text messaging intervention to improve physical activity that delivered messages at an empirically derived decision point, based on prior research indicating that adolescents are likely to exercise between 5 PM and 8 PM [26]. The overarching goal of the study was to refine the intervention and protocol for future trials.

### Principal Findings

Our feasibility (ie, failure to meet recruitment rate but successfully met attrition rate) and acceptability results indicate not only adolescent enthusiasm about the intervention but also potential modifications to improve recruitment for future trials. These results highlight TAGS’s functionality, ease of use, and readiness for future trials. There were no technological problems with the TAGS platform, indicating positive prognosis for the replication of the TAGS messaging system in future trials. The high satisfaction and usability ratings demonstrate user understanding of the intervention with minimal teaching from the investigation team. TAGS shows promise for high scalability, such as recruiting larger samples in future trials and for rapid deployment of the intervention, due to its comprehensible and digital structure [9]. As participants noted, the ease and convenience of the TAGS program to everyday life was a notable strength of the protocol, which is correlated with high engagement [36] and uptake [37] of digital interventions as well as indicative of high implementation [38].

TAGS included several strategies for effective engagement such as personalization of intervention content (eg, providing tailored feedback) and just-in-time adaptation [39]. The engagement results add support for the decision points or intervention timing of the TAGS system and demonstrate the likelihood of sustained engagement in TAGS in future trials. This is based on our findings that engagement was higher in TAGS than in NUDGE [21] and that most participants (7/10, 70%) did not passively withdraw their engagement with the 3 PM message. In addition, based on the qualitative responses, it seems that the participants who permanently disengaged from the 3 PM message had concerns related to device burden as opposed to the TAGS messages. Given the engagement with the 3 PM TAGS message, retaining this decision point for a future iteration of TAGS or NUDGE could further enhance adolescents’ physical activity in the next few hours. However, a future study might use different strategies at this time given the changes to MVPA were minimal.

TAGS sought to enhance the control theory [23,24] and BCT strategies (self-monitoring, feedback) [22,25] embedded within NUDGE by providing quantitative feedback on adolescents’ progress toward their daily physical activity goal at an opportune decision point [26] to yield engagement in these strategies and subsequent activity improvements [15,40]. The exploratory analyses suggest that for teenagers who received an intervention with similar mechanisms (NUDGE), additional engagement in these strategies may not have added physical activity benefits even if at an opportune moment (TAGS). Still, the MVPA change in the TAGS group from preintervention to intervention (approximately 2 minutes) was about half of the magnitude of the MVPA change found by Metcalf and colleagues [7] in their meta-analysis on the efficacy of youth physical activity interventions. It is important to note that most of the interventions in the meta-analysis by Metcalf and coauthors [7] were resource-intensive, in-person interventions that lasted at least 4 weeks compared with the minimalistic, digital, 20-day TAGS intervention. In other words, TAGS was able to produce about half the magnitude of in-person interventions with very low resources and in a short amount of time.

Because most adolescents responded to the TAGS message often, adolescents might benefit from other physical activity promotion strategies during the 3 PM to 8 PM window. Other strategies could include the employment of other BCTs [41], tailoring intervention content to participant characteristics, or incorporating other context-aware approaches. A future iteration of TAGS might include intervention options that address the in-the-moment time contextual predicaments of participants during the 3 PM to 8 PM window. In fact, there are existing digital physical activity interventions that use context-aware sensing to tailor intervention options to changes in users’ contexts. For example, the HeartSteps intervention used contextually tailored suggestions to help users be more physically active or less sedentary based on multiple factors in their current context (eg, location, weather, day of week, time of day) [42]. TAGS participants completed EMA surveys of their mood and affect and wore a GPS device during the study, allowing for future analyses on the proximal affective and location associations with their engagement and physical activity. These analyses will inform what factors might have prevented or promoted engaged and physical activity (eg, such as if a participant was in a location that was not optimal to respond to texts or if a participant was feeling fatigued) and lead to the design of relevant contextually tailored intervention options.

Some degree of nonengagement is expected in digital interventions [13] as exhibited by participants in clusters 2 and 3. Further inquiry into the proximal factors and individual characteristics contributing to nonengagement can help build tailored engagement strategies [15] and inform opportunities to refine the TAGS protocol. For example, adolescents in cluster 2 displayed sporadic patterns of responding but still demonstrated adequate levels of engagement consistent with other adolescent digital interventions [12,21,43]. For these participants, perhaps the 3 PM timing of the TAGS message was not always helpful for them given other competing demands in the participant’s context at that time and other proximal, within-person fluctuations in their affect or psychological state. That is to say that there may be more immediate factors contributing to patterns of engagement such as location changes as well as fluctuations in mood and affect [44]. Enhanced context-aware sensing would provide additional data regarding the user’s contextual and affective state that could then be appropriately leveraged to make decisions regarding optimal decision points beyond just the time of day.

However, participants in cluster 3 passively disengaged from TAGS. Participants in this cluster had a reduced number of wear days for the accelerometer, reported a dislike of completing of the EMA or GPS components of the study as well as wearing the accelerometer, and more negatively rated the study (compared with the other participants) on the TAGS acceptability measure. It appears that these participants may have found the study burdensome, and enhanced intervention support (as outlined for those in cluster 2) would not have relieved this burden. However, these participants may have disengaged due to burden related to completing the overall study protocol but not necessarily a dislike of the TAGS intervention. For example, most participants did not explicitly comment or state concerns about the TAGS intervention. The lack of dislikes about the intervention as opposed to other aspects of the study protocol suggests that reducing study burden might lead to greater levels of engagement in future trials. In addition, this study’s rate of recruitment failed to meet the hypothesized threshold likely due to concerns about anticipated study burden (eg, concerns about wearing the devices). In summary, these findings suggest the need to minimize the burden of the TAGS protocol, such as the overall study requirements, to boost engagement and recruitment in future trials.

### Limitations and Future Directions

The findings of this study should be considered in the context of the study’s limitations such as its small sample and quasi-experimental design, which limits causal inferences. However, this design with a smaller sample was appropriate given the goals of the study [27]. Demographic variables between groups were not perfectly matched and there may be subtle differences across samples due to these factors as well as other individual characteristics, which may impact the internal validity of the efficacy results. A future study would benefit from randomization of participants and balanced recruitment of adolescents across known correlates of MVPA including sex assigned at birth, age, and built environment [45-47] to name a few. Another limitation of the study is small TAGS sample, impacting the generalizability of the findings. There is a possibility of selection bias for the TAGS sample, as these 10 adolescents were willing to complete a month-long study including wearing multiple study devices, completing a physical activity intervention, and attending 3 study visits. In addition, engagement results might be lower for a longer intervention period (>20 days) given increased disengagement from users over time in text message–based digital interventions [12]. A longer trial would need to consider how to keep users engaged despite possible notification fatigue.

A future trial of TAGS should consider using a mixed methods, human-centered design approach [48] to examine participants’ perceptions of TAGS more comprehensively, gauge their ideas for refining TAGS to meet their needs, and qualitatively assessing their reasons for engaging or nonengaging. It is important to work collaboratively *with* adolescents in the design and development of TAGS refinements to equitably address their needs and to include their input [14]. Although a future trial might not include multiple study components (ie, incorporation of EMA or GPS measurements), participants will likely need to wear or use a device to objectively measure their physical activity which will inflict some burden. Given feedback from participants in this study, a future trial might consider using a more inconspicuous, comfortable device with evidence for accurately classifying physical activity to reduce burden.

### Conclusions

The main outcome for this proof-of-concept study is a mostly feasible, acceptable, and technically and functionally reliable digital physical activity intervention with which adolescents engaged. TAGS shows promise for future trials with additional refinements given participants’ engagement with the system and the small MVPA improvements relative to its minimalistic structure. TAGS can be strengthened by reducing burden of the study protocol, such as using less conspicuous and more comfortable measurement devices, and testing other strategies to boost MVPA at the 3 PM decision point, including in-the-moment contextually tailored strategies. Results support that the 3 PM to 8 PM window may be an opportune decision point for a future adolescent digital physical activity JITAI. Further optimization of TAGS platform will prepare the intervention for more rigorous testing.

## Appendix

Multimedia Appendix 1 Additional materials that include study measures.

## Abbreviations

BCT: behavior change technique
CSQ: Consumer Satisfaction Questionnaire
EMA: ecological momentary assessment
ICC: intraclass correlation coefficient
JITAI: Just-in-Time Adaptive Intervention
MVPA: moderate to vigorous physical activity
NUDGE: Network Underwritten Dynamic Goals Engine
ORBIT: Obesity-Related Behavioral Interventions Trials
SUS: System Usability Scale
TAGS: Temporally Augmented Goal Setting

## Data Availability

The datasets generated during and/or analyzed during this study are available from the corresponding author on reasonable request.

## References

[1] Lee IM, Shiroma EJ, Lobelo F, Puska P, Blair SN, Katzmarzyk PT, Lancet Physical Activity Series Working Group (2012). Effect of physical inactivity on major non-communicable diseases worldwide: an analysis of burden of disease and life expectancy. Lancet.

[2] Katzmarzyk PT, Denstel KD, Beals K, Bolling C, Wright C, Crouter SE, McKenzie TL, Pate RR, Saelens BE, Staiano AE, Stanish HI, Sisson SB (2016). Results from the United States of America's 2016 report card on physical activity for children and youth. J Phys Act Health.

[3] Hayes G, Dowd KP, MacDonncha C, Donnelly AE (2019). Tracking of physical activity and sedentary behavior from adolescence to young adulthood: a systematic literature review. J Adolesc Health.

[4] Corder K, Winpenny E, Love R, Brown HE, White M, Sluijs EV (2019). Change in physical activity from adolescence to early adulthood: a systematic review and meta-analysis of longitudinal cohort studies. Br J Sports Med.

[5] Telama R (2009). Tracking of physical activity from childhood to adulthood: a review. Obes Facts.

[6] Cushing CC, Brannon EE, Suorsa KI, Wilson DK (2014). Systematic review and meta-analysis of health promotion interventions for children and adolescents using an ecological framework. J Pediatr Psychol.

[7] Metcalf B, Henley W, Wilkin T (2012). Effectiveness of intervention on physical activity of children: systematic review and meta-analysis of controlled trials with objectively measured outcomes (EarlyBird 54). BMJ.

[8] Cushing CC, Fedele DA, Riley WT (2019). Introduction to the coordinated special issue on eHealth/mHealth in pediatric psychology. J Pediatr Psychol.

[9] Direito A, Carraça E, Rawstorn J, Whittaker R, Maddison R (2017). mHealth technologies to influence physical activity and sedentary behaviors: behavior change techniques, systematic review and meta-analysis of randomized controlled trials. Ann Behav Med.

[10] Psihogios AM, Lane-Fall MB, Graham AK (2022). Adolescents are still waiting on a digital health revolution: accelerating research-to-practice translation through design for implementation. JAMA Pediatr.

[11] Yardley L, Spring BJ, Riper H, Morrison LG, Crane DH, Curtis K, Merchant GC, Naughton F, Blandford A (2016). Understanding and promoting effective engagement with digital behavior change interventions. Am J Prev Med.

[12] Psihogios AM, Li Y, Butler E, Hamilton J, Daniel LC, Barakat LP, Bonafide CP, Schwartz LA (2019). Text message responsivity in a 2-way short message service pilot intervention with adolescent and young adult survivors of cancer. JMIR Mhealth Uhealth.

[13] Eysenbach G (2005). The law of attrition. J Med Internet Res.

[14] Stiles-Shields C, Ramos G, Ortega A, Psihogios AM (2023). Increasing digital mental health reach and uptake via youth partnerships. Npj Ment Health Res.

[15] Nahum-Shani I, Smith SN, Spring BJ, Collins LM, Witkiewitz K, Tewari A, Murphy SA (2018). Just-in-time adaptive interventions (JITAIs) in mobile health: key components and design principles for ongoing health behavior support. Ann Behav Med.

[16] Hardeman W, Houghton J, Lane K, Jones A, Naughton F (2019). A systematic review of just-in-time adaptive interventions (JITAIs) to promote physical activity. Int J Behav Nutr Phys Act.

[17] Bond DS, Thomas JG, Raynor HA, Moon J, Sieling J, Trautvetter J, Leblond T, Wing RR (2014). B-MOBILE—a smartphone-based intervention to reduce sedentary time in overweight/obese individuals: a within-subjects experimental trial. PLoS One.

[18] Pellegrini CA, Hoffman SA, Daly ER, Murillo M, Iakovlev G, Spring B (2015). Acceptability of smartphone technology to interrupt sedentary time in adults with diabetes. Transl Behav Med.

[19] van Dantzig S, Geleijnse G, van Halteren AT (2012). Toward a persuasive mobile application to reduce sedentary behavior. Pers Ubiquitous Comput.

[20] Finkelstein J, Bedra M, Li X, Wood J, Ouyang P (2015). Mobile app to reduce inactivity in sedentary overweight women. Stud Health Technol Inform.

[21] Cushing CC, Bejarano CM, Ortega A, Sayre N, Fedele DA, Smyth JM (2021). Adaptive mHealth intervention for adolescent physical activity promotion. J Pediatr Psychol.

[22] Brannon EE, Cushing CC (2015). A systematic review: is there an app for that? Translational science of pediatric behavior change for physical activity and dietary interventions. J Pediatr Psychol.

[23] Carver CS, Scheier MF (1981). Attention and Self-Regulation?: A Control-Theory Approach to Human Behavior.

[24] Carver CS, Scheier MF (1982). Control theory: a useful conceptual framework for personality-social, clinical, and health psychology. Psychol Bull.

[25] Michie S, Abraham C, Whittington C, McAteer J, Gupta S (2009). Effective techniques in healthy eating and physical activity interventions: a meta-regression. Health Psychol.

[26] Ortega A, Cushing CC (2020). Developing empirical decision points to improve the timing of adaptive digital health physical activity interventions in youth: survival analysis. JMIR Mhealth Uhealth.

[27] Czajkowski SM, Powell LH, Adler N, Naar-King S, Reynolds KD, Hunter CM, Laraia B, Olster DH, Perna FM, Peterson JC, Epel E, Boyington JE, Charlson ME (2015). From ideas to efficacy: the ORBIT model for developing behavioral treatments for chronic diseases. Health Psychol.

[28] Brannon EE, Cushing CC, Walters RW, Crick C, Noser AE, Mullins LL (2018). Goal feedback from whom? A physical activity intervention using an N-of-1 RCT. Psychol Health.

[29] Brannan AM, Sonnichsen SE, Heflinger CA (1996). Measuring satisfaction with children's mental health services: validity and reliability of the satisfaction scales. Eval Program Plann.

[30] Finstad K (2010). The usability metric for user experience. Interact Comput.

[31] Troiano RP, Berrigan D, Dodd KW, Mâsse LC, Tilert T, McDowell M (2008). Physical activity in the United States measured by accelerometer. Med Sci Sports Exerc.

[32] Sadeh A, Sharkey K M, Carskadon M A (1994). Activity-based sleep-wake identification: an empirical test of methodological issues. Sleep.

[33] Chandler JL, Brazendale K, Beets MW, Mealing BA (2016). Classification of physical activity intensities using a wrist-worn accelerometer in 8-12-year-old children. Pediatr Obes.

[34] Bejarano CM, Cushing CC, Crick CJ (2019). Does context predict psychological states and activity? An ecological momentary assessment pilot study of adolescents. Psychol Sport Exerc.

[35] Cohen J (1988). Statistical Power Analysis for the Behavioral Sciences. 2nd ed.

[36] Borghouts J, Eikey E, Mark G, De Leon C, Schueller SM, Schneider M, Stadnick N, Zheng K, Mukamel D, Sorkin DH (2021). Barriers to and facilitators of user engagement with digital mental health interventions: systematic review. J Med Internet Res.

[37] Szinay D, Jones A, Chadborn T, Brown J, Naughton F (2020). Influences on the uptake of and engagement with health and well-being smartphone apps: systematic review. J Med Internet Res.

[38] Graham AK, Lattie EG, Powell BJ, Lyon AR, Smith JD, Schueller SM, Stadnick NA, Brown CH, Mohr DC (2020). Implementation strategies for digital mental health interventions in health care settings. Am Psychol.

[39] Partridge S, Redfern J (2018). Strategies to engage adolescents in digital health interventions for obesity prevention and management. Healthcare (Basel).

[40] Valle CG, Tate DF (2017). Engagement of young adult cancer survivors within a Facebook-based physical activity intervention. Transl Behav Med.

[41] Abraham C, Michie S (2008). A taxonomy of behavior change techniques used in interventions. Health Psychol.

[42] Klasnja P, Smith S, Seewald NJ, Lee A, Hall K, Luers B, Hekler EB, Murphy SA (2019). Efficacy of contextually tailored suggestions for physical activity: a micro-randomized optimization trial of HeartSteps. Ann Behav Med.

[43] Franklin VL, Greene A, Waller A, Greene SA, Pagliari C (2008). Patients' engagement with "Sweet Talk"—a text messaging support system for young people with diabetes. J Med Internet Res.

[44] Sarker H, Sharmin M, Ali AA, Rahman MM, Bari R, Hossain SM, Kumar S (2014). Assessing the availability of users to engage in just-in-time intervention in the natural environment. Proc ACM Int Conf Ubiquitous Comput.

[45] Trost SG, Pate RR, Sallis JF, Freedson PS, Taylor WC, Dowda M, Sirard J (2002). Age and gender differences in objectively measured physical activity in youth. Med Sci Sports Exerc.

[46] Ding D, Sallis JF, Kerr J, Lee S, Rosenberg DE (2011). Neighborhood environment and physical activity among youth a review. Am J Prev Med.

[47] Sallis JF, Conway TL, Cain KL, Carlson JA, Frank LD, Kerr J, Glanz K, Chapman JE, Saelens BE (2018). Neighborhood built environment and socioeconomic status in relation to physical activity, sedentary behavior, and weight status of adolescents. Prev Med.

[48] Stiles-Shields C, Cummings C, Montague E, Plevinsky JM, Psihogios AM, Williams KDA (2022). A call to action: using and extending human-centered design methodologies to improve mental and behavioral health equity. Front Digit Health.

