# Supplementary material for: Design of a Temporally Augmented Text Messaging Bot to Improve Adolescents’ Physical Activity and Engagement: Proof-of-Concept Study
Source: JMIR Form Res. 2024 Oct 10;8:e60171. doi: 10.2196/60171 (PMC11502983; doi:10.2196/60171)
Supplement: Multimedia Appendix 1 [file formative_v8i1e60171_app1.docx]

**TAGS Satisfaction Questionnaire (CSQ)**

Please help us improve TAGS by answering some questions about the services you receive. We are interested in your honest opinions, whether they are positive or negative.

|  | Excellent | Good | Fair | Poor |
| --- | --- | --- | --- | --- |
| How do you rate the overall quality with TAGS? | 1 | 2 | 3 | 4 |
|  | No, definitely not | No, not really | Yes, generally | Yes, definitely |
| Did you get helpful services from TAGS? | 1 | 2 | 3 | 4 |
|  | TAGS has been extremely helpful | TAGS has been mostly helpful | TAGS has not been helpful | TAGS has been unhelpful |
| To what extent has TAGS helped your exercise? | 1 | 2 | 3 | 4 |
|  | No, definitely not | No, not really | Yes, generally | Yes, definitely |
| If a friend were in need of help improving their exercise, would you recommended TAGS to them? | 1 | 2 | 3 | 4 |
|  | Quite dissatisfied | Indifferently or mildly dissatisfied | Mostly satisfied | Very Satisfied |
| How satisfied are you with the amount of help TAGS gave you? | 1 | 2 | 3 | 4 |
|  | Yes, it helped a great deal | Yes, it somewhat helped | No, it really didn’t help | No, it seemed to make things worse |
| Has TAGS helped you improve your exercise? | 1 | 2 | 3 | 4 |
|  | Very Satisfied | Mostly satisfied | Indifferently or mildly dissatisfied | Quite dissatisfied |
| In general, how satisfied are you with TAGS? | 1 | 2 | 3 | 4 |
|  | No, definitely not | No, not really | Yes, generally | Yes, definitely |
| If you were to seek help with your exercise in the future, would you use TAGS again? | 1 | 2 | 3 | 4 |

**TAGS System Usability Scale (SUS)**

1: I think that I would like to use the TAGS messaging system frequently

| 1 | 2 | 3 | 4 | 5 |
| --- | --- | --- | --- | --- |
| Strongly disagree |  |  |  | Strongly agree |

2: I found the TAGS messaging system unnecessarily complex

| 1 | 2 | 3 | 4 | 5 |
| --- | --- | --- | --- | --- |
| Strongly disagree |  |  |  | Strongly agree |

3: I think the TAGS messaging system was easy to use

| 1 | 2 | 3 | 4 | 5 |
| --- | --- | --- | --- | --- |
| Strongly disagree |  |  |  | Strongly agree |

4: I think that I would need the support of a technical person to be able to use the TAGS messaging system

| 1 | 2 | 3 | 4 | 5 |
| --- | --- | --- | --- | --- |
| Strongly disagree |  |  |  | Strongly agree |

5: I found the various functions of the TAGS messaging system were well integrated

| 1 | 2 | 3 | 4 | 5 |
| --- | --- | --- | --- | --- |
| Strongly disagree |  |  |  | Strongly agree |

6: I thought there was too much inconsistency in this TAGS messaging system

| 1 | 2 | 3 | 4 | 5 |
| --- | --- | --- | --- | --- |
| Strongly disagree |  |  |  | Strongly agree |

7: I would imagine that most people would learn to use the TAGS messaging system very quickly

| 1 | 2 | 3 | 4 | 5 |
| --- | --- | --- | --- | --- |
| Strongly disagree |  |  |  | Strongly agree |

8: I found the TAGS messaging system very cumbersome to use

| 1 | 2 | 3 | 4 | 5 |
| --- | --- | --- | --- | --- |
| Strongly disagree |  |  |  | Strongly agree |

9: I felt very confident using the TAGS messaging system

| 1 | 2 | 3 | 4 | 5 |
| --- | --- | --- | --- | --- |
| Strongly disagree |  |  |  | Strongly agree |

10: I needed to learn a lot of things before I could get going with the TAGS messaging system

| 1 | 2 | 3 | 4 | 5 |
| --- | --- | --- | --- | --- |
| Strongly disagree |  |  |  | Strongly agree |

**TAGS Acceptability Questionnaire**

We are interested in understanding your experience of the TAGS progrram. We want to know things you liked and things you did not like. As it relates to the heart rate monitor, wrist strap, and questionnaires on the smartphone please answer the following questions:

1. I enjoyed participating in the study.

1 2 3 4 5 6 7

Not at Somewhat Very

all true true true

2. I thought the wrist strap was comfortable to wear each day for 20 days.

1 2 3 4 5 6 7

Not at Somewhat Very

all true true true

3. Answering the phone surveys was easy.

1 2 3 4 5 6 7

Not at Somewhat Very

all true true true

4. Answering the phone surveys took too much time.

1 2 3 4 5 6 7

Not at Somewhat Very

all true true true

5. If my doctor asked me to do a study like this to know more about my health I would do it.

1 2 3 4 5 6 7

Not at Somewhat Very

all true true true

6. In your own words, please tell us what you liked about the study.

7. In your own words, please tell us what you did not like about the study.
